# Biomechanical comparison of tendon repair techniques: Bunnell suture leads in mode of failure and minimally invasive configuration in elongation

**DOI:** 10.1002/jeo2.70236

**Published:** 2025-04-17

**Authors:** Umile Giuseppe Longo, Stefano Campi, Martina Marino, Margaux D'Hooghe, Maristella Saccomanno, Kristian Samuelsson, Francisco Forriol, Vincenzo Denaro

**Affiliations:** ^1^ Fondazione Policlinico Universitario Campus Bio‐Medico Roma Italy; ^2^ Department of Medicine and Surgery Research Unit of Orthopaedic and Trauma Surgery, Università Campus Bio‐Medico di Roma Roma Italy; ^3^ Department of Medicine University of Navarra Pamplona Spain; ^4^ Department of Medical and Surgical Specialties, Radiological Sciences, and Public Health University of Brescia Brescia Italy; ^5^ Department of Bone and Joint Surgery Speciali Civili Brescia Italy; ^6^ Sahlgrenska Sports Medicine Center Gothenburg Sweden; ^7^ Department of Orthopaedics Institute of Clinical Sciences, Sahlgrenska Academy University of Gothenburg Gothenburg Sweden; ^8^ Department of Orthopaedics Sahlgrenska University Hospital Mölndal Sweden; ^9^ Orthopaedic Surgery Department University Foundation San Pablo CEU Madrid Spain

**Keywords:** Bunnel suture, Kessler suture, minimally invasive suture configuration, percutaneous tendon repair

## Abstract

**Purpose:**

In this experimental study, the Minimally Invasive Configuration (MIC), the Bunnell, Kessler and modified Bunnell–Kessler techniques for tendon rupture repair were compared in terms of the following biomechanical parameters: maximum load, mode of failure, failure elongation, tension/construct elongation and stiffness (Young's modulus). The scope of comparison involves understanding the properties of each suture technique in hopes of eventually contributing to surgical decision‐making.

**Methods:**

Thirty‐two frozen ovine specimens were obtained, and transverse tenotomy was performed on each. Eight tendons were randomly allocated to each technique. Specimens were tested performing a unidirectional tensile load to failure using a servo‐hydraulic testing device. The tendons were also loaded to failure at a rate of 10 mm/s. The total length of the construct was defined as the distance from the two clamps. Stiffness was calculated by determining the slope of the force–displacement curve in the linear region. Total failure was defined as a drop of measured force or rupture of the tendon–suture complex.

**Results:**

In the mode of failure, Tukey's post hoc test showed a statistically significant difference between the Bunnell group and the other three groups (*p* < 0.05). For Tension/construct elongation at 5 and 10 mm, Tukey's post hoc test showed a statistically significant difference between the MIC group and the other three groups (*p* < 0.05). At 15 mm Tukey's post hoc test showed a statistically significant difference between the MIC group and the Kessler group (*p* < 0.05).

**Conclusion:**

The Bunnell suture performed best in terms of mode of failure, while the MIC suture technique outperformed the rest in terms of tension/construct elongation. Findings show sufficient biomechanical evidence to support the ongoing clinical application of all techniques.

**Level of Evidence:**

Level V.

**Abbreviations**
ANOVA: analysis of variance
AT: Achilles tendon
MIC: minimally invasive configuration

## INTRODUCTION

Tendon injuries and lacerations are common occurrences in the orthopaedic field [26, 32]. Injuries range from traumatic flexor tendon lacerations [35] to sports‐related accidents causing Achilles tendon (AT) ruptures [7, 34, 35]. Due to their high incidence, high‐quality suture techniques that aim to provide maximum mechanical stability are crucial for recovery [6, 9, 17, 21, 24, 33, 36]. The mechanical stability of a repaired tendon depends on two variables: the tensile strength of the material used in repair, and the tendon‐holding capacity of the technique [18, 22, 27]. Additionally, repair should reduce the incidence of reruptures and allow early rehabilitation and active mobilization [6, 9, 17, 21, 24, 33, 36].

Various open and, more recently, percutaneous approaches have been developed for the repair of tendon rupture, each with its own biomechanical advantages and disadvantages. The minimally invasive configuration (MIC), modelled after the suture that is placed using the Achillon device (Achillon; Wright Medical) [2], a mini‐open percutaneous method of tendon repair, has only been tested in a few biomechanical studies resulting in controversial outcomes [8, 10, 19]. The Bunnell suture is an interlacing suture which creates a locking stitch that provides strong and stable tendon repair and, due to its effectiveness, it is widely regarded as the ‘gold standard’ for open AT repair [4, 11]. Kessler repair is characterized by interlacing the tendon with a large contact area between the tendon and the thread surface, which provides high stability and promotes effective healing [13]. Maximizing biomechanical strength is often combined with other methods, such as the Bunnell or Krakow techniques, to achieve optimal results [13]. Hence, the development of the Bunnell–Kessler technique is believed to better distribute tension along the length of the tendon, reducing the risk of re‐rupture [8, 23].

Individually, each of these techniques is reported to have good mechanical strength providing optimal outcomes for resistant tendon repair, however, literature regarding the biomechanical comparison of each is still lacking [1, 2, 8]. Knowing the different biomechanical properties of these sutures, and as a result understanding their advantages and disadvantages, can aid in the process of case‐by‐case selection of the appropriate suture technique. The latter must provide sufficient mechanical strength and stability to allow patients to return to daily activities and sports, while also maintaining long‐term repair with low risk of re‐rupture.

Thus, in this experimental study, the MIC, the Bunnell, Kessler and modified Bunnell–Kessler techniques for tendon rupture repair were compared in terms of the following biomechanical parameters: maximum load, mode of failure, failure elongation, tension/construct elongation and stiffness (Young's modulus).

## MATERIALS AND METHODS

### Ethical approval

Approval for the study protocol was granted by the Committee for Animal Experimentation of our hospital and followed the current regulations in accordance with international law on experimentation with animals [14]. The registration number assigned to the protocol is 005/2010.

### Experimental protocol

Eight ovine frozen flexor digitorum tendons were obtained from experimental animals for each group yielding a total of 32 specimens. Sharp dissection, for separation of the Tendons from surrounding muscle and bone tissue, was performed within the first 2 h after the animals were killed and frozen. Prior to use, tendons were defrosted overnight in a refrigerator at 4°C. The specimens were kept moist with saline solution at room temperature throughout testing.

A priori power analysis indicated that a total sample size of 32 specimens (8 per group) was required to achieve statistical significance at a 0.05 level with 80% power.

Antero‐posterior and transverse diameters were measured using a digital calliper (Scienceware Vernier Direct Reading Calipers model H13415‐0000, Bel‐Art Products). Three measurements were taken for each tendon, and the mathematical average was used for statistical purposes.

A transverse tenotomy was performed with a number 10 scalpel (Bard‐Parker). A drawing was used to guide repair and to ensure that all sutures were passed at the same distance through the tendon.

Tendons were randomly allocated to one of the four techniques. All the sutures were performed by the same surgeon. The material used in all the techniques was No. 2‐0 Prolene sutures (Ethicon) with a 30 mm round‐bodied needle. In all groups, the sutures were fixed with five surgeon's knots.

**Figure 1:**
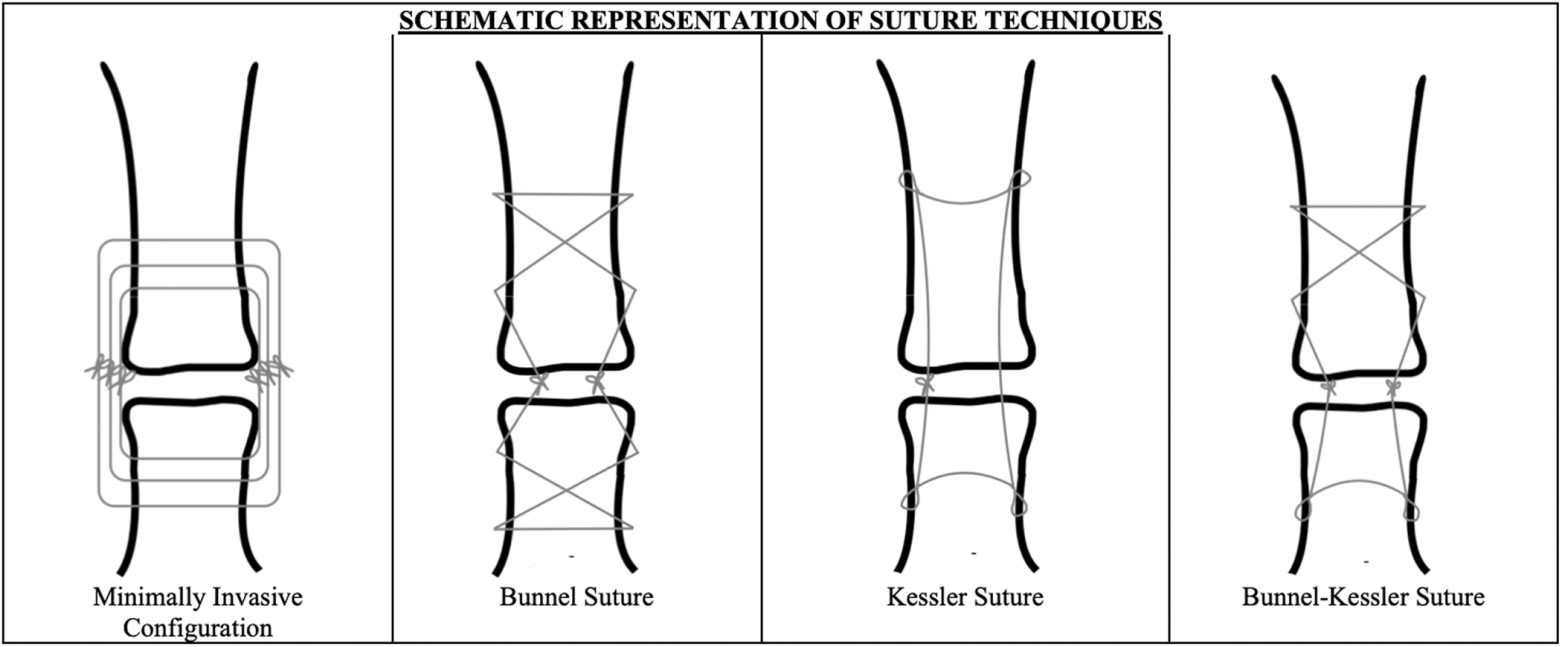
Schematic representation of suture techniques. MIC, minimally invasive configuration.

The following techniques were adopted for each group (graphical representation can be seen in Figure 1):

− In the MIC group, tendons were repaired with three concentric sutures, reproducing the Achillon device suture configuration system (Achillon; Wright Medical), as described by Lee et al. [16].
− In the Bunnell group, tendons were repaired according to the suture configuration proposed by Bunnell [4].
− In the Kessler group, tendons were repaired according to suture configuration described by Kessler [13].
− In the Bunnell–Kessler group, tendons were repaired using the Bunnell suture configuration on the proximal side and the Kessler scheme on the distal side of the construct.

After the tendons were sutured, they were preloaded to 5 N for 2 min. Specimens were tested performing a unidirectional tensile load to failure using a servo‐hydraulic testing device (MTS Systems) controlled by an Electropuls e3000 Instron (Instron Ltd.). The tendon was secured, with a pressure of 5 atm, proximally and distally onto pneumatic clamps such that the free length was standardized across the specimens. The tendons were then loaded to failure at a rate of 10 mm/s. The total length of the construct was defined as the distance from the two clamps [31].

Failure loads and axial displacements were measured directly by the Instron machine and digitally transferred to a computer.

Stiffness was calculated by determining the slope of the force–displacement curve in the linear region [12, 31]. Total failure was defined as a precipitous drop of measured force or as a rupture of the tendon–suture complex [37].

### Statistical analysis

The analysis consisted of descriptive means and standard deviations (SDs) for continuous outcome measures. Inferential analysis was performed using one‐way analysis of variance (ANOVA) comparing each of the independent groups with the dependent outcomes of interest, which included maximum load, failure elongation, stiffness (Young's module) and tension value at 5, 10, 15 and 20 mm of elongation. If the overall difference was significant, multiple comparisons were performed between groups using Tukey's post hoc test. *p* < 0.05 was considered significant.

## RESULTS

### Maximum load

The mean maximum load was 48.86 N (SD = 11.58) for the MIC group, 48.7 N (SD = 6.05) for the Bunnell group, 37.5 N (SD = 15.44) for the Kessler group and 45.02 N (SD = 12.88) for the Bunnell–Kessler group. The ANOVA showed no statistically significant difference between the four groups (*p* = 0.22) (Table 1).

**Table 1:** Reported mean maximum load, mean failure elongation and stiffness (Young's module).

	MIC	Bunnell	Kessler	Bunnell–Kessler
Mean maximum load (N; SD)	48.86 (SD = 11.58)	48.7 (SD = 6.05)	37.5 (SD = 15.44)	45.02 (SD = 12.88)
Mean failure elongation (mm; SD)	22.22 (SD = 8.16)	26.4 (SD = 4.75)	23.87 (SD = 7.0)	26.91 (SD = 4.78)
Stiffness (N/mm; SD)	6.52 N/mm (SD = 1.99)	3.44 N/mm (SD = 0.63)	4.84 N/mm (SD = 3.02)	4.88 N/mm (SD = 2.51)

Abbreviation: SD, standard deviation.

### Failure elongation

The mean failure elongation was 22.22 mm (SD = 8.16) for the MIC group, 26.4 mm (SD = 4.75) for the Bunnell group, 23.87 mm (SD = 7.0) for the Kessler group and 26.91 mm (SD = 4.78) for the Bunnell–Kessler group. The ANOVA showed no statistically significant difference between the four groups (*p* = 0.42) (Table 1).

### Stiffness (Young's modulus)

The mean stiffness of the tendon–suture complex was 6.52 N/mm (SD = 1.99) for the MIC group, 3.44 N/mm (SD = 0.63) for the Bunnell group, 4.84 N/mm (SD = 3.02) for the Kessler group and 4.88 N/mm (SD = 2.51) for the Bunnell–Kessler group. The ANOVA showed no statistically significant difference between the four groups (p = 0.075) (Table 1).

### Mode of failure

In the MIC group, all sutures failed by suture pull‐out through the tendon. In the Bunnell group, one suture failed by suture pull‐out through the tendon, and seven failed by suture breakage. In the Kessler group, six sutures failed by suture pull‐out through the tendon, and two failed by suture breakage. In the Bunnell–Kessler group, six sutures failed by suture pull‐out through the tendon, and two failed by suture breakage. The pull‐out of the suture in this group always occurred on the Kessler‐like side. The ANOVA showed a statistically significant difference between the four groups (*p* = 0.05). Tukey's post hoc test showed a statistically significant difference between the Bunnell group and the other three groups (p = 0.04) (Table 2).

**Table 2:** Reported mode of failure.

Mode of failure (no.)		
	Suture pull‐out	Suture breakage
MIC	8	0
Bunnell	1	7
Kessler	6	2
Bunnell–Kessler	6^a^	2

Abbreviation: MIC, minimally invasive configuration.

a Always occurred on Kessler's side.

### Tension/construct elongation

The tension was measured at 5, 10, 15 and 20 mm of construct elongation.

The mean tension value at 5 mm of construct elongation was 18.49 N (SD = 7.76) for the MIC group, 10.03 N (SD = 2.46) for the Bunnell group, 9.53 N (SD = 4.57) for the Kessler group and 11.15 N (SD = 4.19) for the Bunnell–Kessler group. The ANOVA showed a statistically significant difference between the four groups (*p* = 0.005). Tukey's post hoc test showed a statistically significant difference between the MIC group and the other three groups (p = 0.03).

The mean tension value at 10 mm of construct elongation was 34.23 N (SD = 7.91) for the MIC group, 17.81 N (SD = 4.08) for the Bunnell group, 15.82 N (SD = 8.59) for the Kessler group and 21.8 N (SD = 6.59) for the Bunnell–Kessler group. The ANOVA showed a statistically significant difference between the four groups (*p* = 0.005). Tukey's post hoc test showed a statistically significant difference between the MIC group and the other three groups (*p* = 0.01).

The mean tension value at 15 mm of construct elongation was 38.79 N (SD = 13.31) for the MIC group, 26.82 N (SD = 5.18) for the Bunnell group, 24.32 N (SD = 11.02) for the Kessler group and 30.59 N (SD = 9.28) for the Bunnell–Kessler group. The ANOVA showed a statistically significant difference between the four groups (*p* = 0.042). Tukey's post hoc test showed a statistically significant difference between the MIC group and the Kessler group (*p* = 0.04).

**Figure 2:**
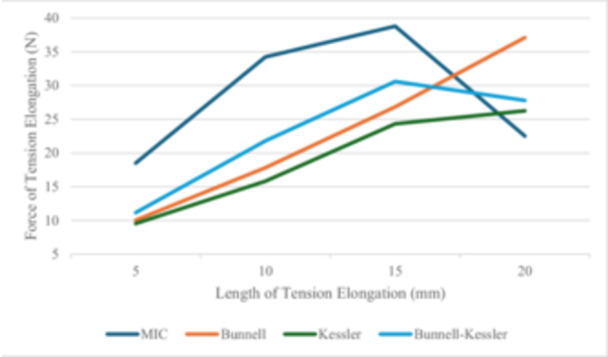
Graph of mean tension/construct elongation at 5, 10, 15 and 20 mm.

The mean tension value at 20 mm of construct elongation was 22.51 N (SD = 22.69) for the MIC group, 37.1 N (SD = 5.42) for the Bunnell group, 26.25 N (SD = 17.47) for the Kessler group and 27.79 N (SD = 21.07) for the Bunnell–Kessler group. The ANOVA showed no statistically significant difference between the four groups (*p* = 0.43) (Table 3, Figure 2).

**Table 3:** Reported mean tension/construct elongation at 5, 10, 15 and 20 mm.

Tension/construct elongation (N; SD)				
(mm)	MIC	Bunnell	Kessler	Bunnell–Kessler
5	18.49 (SD = 7.76)	10.03 (SD = 2.46)	9.53 (SD = 4.57)	11.15 (SD = 4.19)
10	34.23 (SD = 7.91)	17.81 (SD = 4.08)	15.82 (SD = 8.59)	21.8 (SD = 6.59)
15	38.79 (SD = 13.31)	26.82 (SD = 5.18)	24.32 (SD = 11.02)	30.59 (SD = 9.28)
20	22.51 (SD = 22.69)	37.1 (SD = 5.42)	26.25 (SD = 17.47)	27.79 (SD = 21.07)

Abbreviations: MIC, minimally invasive configuration; SD, standard deviation.

## DISCUSSION

Statistically significant findings reveal that the Bunnell suture performs best in terms of mode of failure, while the MIC suture outperformed all other suture techniques in terms of tension elongation.

Seven of the eight trials testing the Bunnell technique found the mode of failure to be due to suture breakage rather than pull‐out through the tendon, highlighting a higher biomechanical stability in terms of tendon holding capacity. Concordantly, a cadaveric study found that when comparing the Bunnell and Kessler techniques, the former had a cord pull‐out, which occurred at an average force of 291 N, while in the latter, this occurred at a force of 180 N [8]. In another cadaveric study, the maximum load was 197 N in the Bunnell group and 101 N in the Kessler group, further displaying the superior strength and resistance to tendon pull‐out failure of the Bunnell suture [11]. Concordantly, our results also showed that when the Kessler–Bunnell technique was utilized, the pull‐out of the suture always occurred on the Kessler‐like side. Despite the difference in tendon origin and suture materials utilized in the mentioned cadaveric studies, the outcome of suture failure was found to be the same, suggesting that the Bunnell technique is likely to provide its maximum strength if a sufficiently loadable suture material is selected [8].

A possible explanation as to why there is a difference in mode of failure is because the Kessler method employs a horizontal rectangle configuration for tendon grasping, using a single suture loop on each side. In contrast, the Bunnell technique creates numerous points of transverse and oblique compression at the intersections of the suture and tendon [38]. This dissimilarity in configuration is likely responsible for the inferior tendon‐holding capacity of the Kessler technique [8, 38]. Nevertheless, the Kessler suture is often applied to flexor tendon repair, highlighting that perhaps its inferior strength is not necessarily a weakness, as the application of its properties and its success is also determined by what kind of repair the suture is applied to [38]. Unfortunately, a documented limitation of the Bunnell suture is that, due to the numerous points of compression employed, there is an element of transverse compression of the tendon, which has been shown to compromise microcirculation at the expense of the healing process and longevity of the repair [11]. As previously mentioned, the in vitro design of this study did not permit the evaluation of this parameter.

When evaluating Tension elongation, a significant difference was found between the MIC group and all other groups. The minimally invasive suture group was found to better withstand tension elongation, a property that, when applied to AT repair, for example, can reduce the likelihood of ruptures and prevent claudication and difficulty in heel‐raising [5]. Despite the good performance of the MIC in the present study, the literature is discordant on its resistance to Tension elongation [6, 20, 31]. In one study, it was found that open repair sutures on cadaveric specimens have significantly less elongation than percutaneous sutures, although their ultimate strength (measured via cycles to failure) was comparable [5]. Furthermore, the same study showed that the application of an open technique for AT rupture repair resulted in considerably less early repair elongation when compared to minimally invasive percutaneous methods during a simulated rehabilitation protocol [5]. Another study comparing various percutaneous approaches found that those applying a locking suture, which is not the case for the MIC, had better strength of construction under cyclic and ultimate loads, although no specific differences were identified in terms of Tension elongation [30].

The greater Tension elongation identified for the MIC suture in the present study is attributed to a progressive failure of the sutures; when a first suture fails, the second still holds the tendon ends opposed to one another until gradual failure of all three concentric sutures. Therefore, although the overall Tension elongation may be higher, it is simply due to a progressive suture‐failure, and it is therefore not an advantageous property of this technique. Furthermore, this configuration utilizes six knots to secure the sutures. On the one hand, this may ensure decreased rates of failure, but on the other, it introduces points that may compromise the microcirculation of the tendon [19, 25, 38]. The present finding alone, in addition to the contrasting literature, is not enough to fully ascertain that the MIC suture, when applied in vivo, has the highest Tension elongation compared to the other techniques viewed.

No statistically significant differences were found in Stiffness, Failure elongation, and Maximum load across suture techniques. Overall, despite some differences in performance, there seems to be sufficient biomechanical evidence to support the ongoing clinical application of all techniques [1, 9, 11, 20, 29, 34].

The first limitation of this study was the use of ovine cadaveric specimens, which do not hold the same tensile properties as human tendons [20]. For example, their spiral pattern is believed to contribute to a different mode of failure during rupture [20]. Nevertheless, given the study aim, which focused on comparison of the biomechanical properties of suture techniques, the consistent use of ovine tendons makes results comparable to one another.

A second limitation of the study design is that tendon rupture was simulated surgically with a sharp blade, which differs significantly from ruptured tissue in vivo. Transverse tenotomy does not provide a transferable model to tendon ruptures extending over large segments with fibre disruption proximal and distal to the main site of injury. Despite this, there are potential benefits to utilizing this technique when evaluating biomechanical parameters alone, for example a surgical transverse tenotomy enables the standardization of suture which would not be replicable in an in vivo model. Additionally, transverse tenotomy can be akin to lacerations of the flexor tendons due to cutting‐related injuries. Flexor tendon transection, for example, is responsible for up to 20% of emergency room visits and restoration of function following an unsuccessful repair, which continues to pose significant clinical and surgical challenges in the field of hand surgery [3, 15]. Among other factors, the selection of an appropriate suture technique can influence repair success.

Thirdly, this method of evaluation does not account for the degree of compromise of tendon microcirculation occurring due to the applied suture technique. Increased blood flow in early tendon healing is associated with better long‐term postoperative functional outcomes [28, 29]. Thus, given that the present study only accounts for biomechanical properties, a possible compromise of microcirculation caused by the applied suture techniques was not considered but deserves future investigation.

Finally, it is important to consider that results were recorded at time zero, whereas in clinical practice, the repair is often stabilized in a plaster cast for a variable number of weeks before attempting mobilization [23]. Hence, time‐zero mechanical testing does not replicate real‐life clinical management, which is a parameter that must be considered when applying present findings to suture selection.

## CONCLUSION

The Bunnell suture technique performed best in terms of mode of failure. The MIC suture instead outperformed the rest in terms of construct/failure elongation; however, this was found to be caused by a progressive failure of concentric sutures. The four techniques did not differ in the other biomechanical parameters being assessed, namely stiffness, failure elongation and maximum load. Both Bunnell and MIC raise concerns in terms of the compromise of microcirculation of the tendon. Hence, further research, particularly in vivo, is necessary to evaluate the clinical outcomes of tendon repair when applying these techniques. Despite some differences in performance, there seems to be sufficient biomechanical evidence to support the ongoing clinical application of all techniques.

## AUTHOR CONTRIBUTIONS

**Umile Giuseppe Longo**: Conceptualization; methodology; validation; writing—review and editing; supervision; project administration. **Vincenzo Denaro**: Conceptualization; validation. **Francisco Forriol**: Methodology; data curation. **Stefano Campi**: Methodology, writing—original draft preparation; visualization. **Martina Marino**: Formal analysis; investigation; writing—original draft preparation. **Margaux D'Hooghe**: Data curation. **Maristella Saccomanno**: Supervision. **Kristian Samuelsson**: Project administration. All authors have read and agreed to the published version of the manuscript.

## CONFLICT OF INTEREST STATEMENT

Kristian Samuelsson is a Member of the Board of Directors of Getinge AB (publ) and medtech advisor to Carl Bennet AB. The other authors declare no conflicts of interest.

## ETHICS STATEMENT

The study had the approval of the Committee for Animal Experimentation of FREMAP (ID 005/10) and followed the current regulations, which were in accordance with international law on experimentation with animals.

## Data Availability

The data presented in this study are available on request from the corresponding author.
